# Advances in Radionuclide Therapies for Patients with Neuro-endocrine Tumors

**DOI:** 10.1007/s11912-024-01521-w

**Published:** 2024-04-10

**Authors:** Denise S. Hoogenkamp, Linda J. de Wit–van der Veen, Daphne M. V. Huizing, Margot E. T. Tesselaar, Rachel S. van Leeuwaarde, Marcel P. M. Stokkel, Marnix G. E. H. Lam, Arthur J. A. T. Braat

**Affiliations:** 1 Department of Nuclear Medicine, Netherlands Cancer Institute, Amsterdam, The Netherlands; 2 ENETS Center of Excellence NKI-AVL, The Netherlands Cancer Institute/UMC Utrecht, Amsterdam, The Netherlands; 3 Department of Gastrointestinal Oncology, Netherlands Cancer Institute, Amsterdam, The Netherlands; 4 Department of Endocrinology, University Medical Center Utrecht, Utrecht, The Netherlands; 5 Department of Radiology and Nuclear Medicine, University Medical Center Utrecht, 3584 CX Utrecht, The Netherlands

**Keywords:** Neuroendocrine tumor, Peptide receptor radionuclide therapy, Combination therapy, Radioembolization

## Abstract

**Purpose of Review:**

To provide insights into the role of peptide receptor radionuclide therapy (PRRT) in patients with advanced neuroendocrine tumors (NET) and an overview of possible strategies to combine PRRT with locoregional and systemic anticancer treatments.

**Recent Findings:**

Research on combining PRRT with other treatments encompasses a wide variety or treatments, both local (transarterial radioembolization) and systemic therapies, chemotherapy (i.e., capecitabine and temozolomide), targeted therapies (i.e., olaparib, everolimus, and sunitinib), and immunotherapies (e.g., nivolumab and pembrolizumab). Furthermore, PRRT shows promising first results as a treatment prior to surgery.

**Summary:**

There is great demand to enhance the efficacy of PRRT through combination with other anticancer treatments. While research in this area is currently limited, the field is rapidly evolving with numerous ongoing clinical trials aiming to address this need and explore novel therapeutic combinations.

## Introduction

Neuroendocrine tumors are a broad class of malignancies that can be omnipresent throughout the body but are most commonly found in the gastro-intestinal tract (GEP-NET) and lung. Although regularly classified as rare malignancy, their incidence is increasing worldwide due to an increased awareness and improved diagnostic capabilities to detect NET lesions. Surgical resection of the primary tumor and regional lymph nodes is still the only curative treatment; however, more than 40% of patients initially present with metastatic and advanced disease. At this stage, the disease is considered non-curative, so treatment focusses on controlling tumor volume, reduce tumor related side-effects, improve quality of live (QoL), and prolong survival [1, 2]. In patients with advanced well-differentiated somatostatin-receptor (SSTR) positive (functional) NETs, somatostatin analogs (SSA) are generally recommended as first-line treatment. In patients who progress on SSA, there are a number of systemic therapies that can be considered, including cytotoxic chemotherapy, multityrosine kinase inhibitors (mTKI), mammalian target of rapamycin inhibitors (mTORi), or peptide receptor radionuclide therapy (PRRT) [3, 4].

PRRT has been under development for over two decades as therapy for patients with advanced (metastatic) SSTR-positive tumors. PRRT is a form of systemic radiotherapy that relies on accumulation of a radiolabeled molecules in a cell. This local (internal) irradiation of cells will lead to irreversible damage to sensitive cellular structures, such as mitochondria or DNA, followed by apoptosis. Despite extensive data on disease control using PRRT in (retrospective) clinical cohorts, therapeutic efficacy over standard-of-care (SSA) was proven in the phase 3 randomized controlled trial, NETTER-1 [5•]. The benefit in QoL and progression-free survival after PRRT plus SSA over high-dose SSA-alone led to regulatory approvals and implementation of PRRT into earlier treatment lines in the recent guidelines [2, 6].

While various systemic therapies can be considered for patients with advanced (metastatic) NET, only limited phase 2–3 studies offer a head-to-head comparison of treatments. Additionally, clinical evidence on potential synergistic effects associated with combining treatments is scarce. This review will provide insights into the current role of PRRT in patients with advanced NET and an overview of possible strategies to combine PRRT with locoregional and systemic anticancer treatments that have been described in literature.

## Peptide Targeted Radionuclide Therapies

Ideal for therapeutic radiopharmaceuticals is to have a cellular target that is highly overexpressed on malignant cells and with limited or absent expression in healthy tissues. For NETs, two molecules were initially developed: metaiodobenzylguanidine (mIBG; norepinephrine analog) and octreotide (one of the SSAs) [7–10]. The role of mIBG in patients with GEP-NET has subsided over the years due to its less favorable tumor targeting, toxicity profile and pharmacokinetic behavior, as compared to PRRT [11].

The first PRRT involved the use high-activity [^111^Indium]In-DTPA^0^-octreotide ([^111^In]In-pentetreotide) in 1994 [12, 13]. This radiopharmaceutical, widely utilized for diagnostic imaging, primarily has γ-emissions, but limited emission of (Auger/conversion-)electrons that can induce the therapeutic effects (see Table 1). Advances in the past decade led to the development of improved SSAs with increased affinity for specific subtypes of somatostatin receptors (SSTR), thereby improving tumor targeting and reducing accumulation in healthy tissues. Together with the introduction of novel bifunctional chelating agents (enabling stable binding of therapeutic radiometals; e.g., Yttrium-90 (^90^Y) or Lutetium-177 (^177^Lu)), this has led to the production of radiopharmaceuticals with superior characteristics (e.g. DOTATOC and DOTATATE). These radiopharmaceuticals had high tumor affinity, limited accumulation in non-target organs, high stability of the complex in vivo, and flexibility to label either diagnostic or therapeutic nuclides to similar targeting molecules [7].

**Table 1:** Commonly used radionuclides for imaging and therapy

Radionuclide	Half-life (h)	Main emission	Energy of emission (keV)
^99m^Tc	6.01	*γ*	140
^111^In	67.4	*γ*	172
^18^F	1.8	*β* +	634
^68^ Ga	1.1	*β* +	1190
^90^Y	64.1	*β* −	2284
^177^Lu	160.8	*β* − /*γ*	497/208*
^166^Ho		*β* − /*γ*	1770–1850/81*
^225^Ac	238	*α*	5830

*Highest yields

[^90^Y]Y-DOTATOC should have improved tumor response compared to [^111^In]In-pentetreotide, since initial simulations showed that the median absorbed dose to tumors could be ten times higher compared to ^111^In. In addition, bone marrow toxicity should be less as the simulations showed substantially lower absorbed doses to the red marrow for [^90^Y]Y-DOTATOC (factor 3.5). The major benefit of ^90^Y is its high beta-energy (*E*_max_ 2.28 MeV) and long physical half-life (*T*_1/2_ 64 h), allowing for longer penetration depths in tissue and longer irradiation, thus causing more cellular damage as compared to ^111^In [10, 14]. Despite being generally well tolerated, [^90^Y]Y-DOTATOC therapy’s increased penetration depth and significant retention in the proximal renal tubules may lead to considerable absorbed renal dose and an increased risk of developing (late) renal toxicities [15–17]. Unfortunately, phase-3 studies are still lacking.

The subsequent promising analog to be developed was ^177^Lutetium-labeled DOTATATE ([^177^Lu]Lu-DOTATATE), which has a six- to nine-fold higher affinity for SSTR_2_ and a longer tumor retention time compared to DOTATOC [18]. As a result, the radiation dose to kidneys and red marrow is far less for [^177^Lu]Lu-DOTATATE, with a median of 0.9–1 Gray per gigabecquerel administered activity (Gy/GBq) and 0.02–0.08 Gy/GBq, versus 1.7–6.1 Gy/GBq and 0.3–0.17 Gy/GBq for [^90^Y]Y-DOTATOC respectively [19, 20]. The absorbed dose to the tumor lesions shows similar results, 3.4–10 Gy/GBq for [^177^Lu]Lu-DOTATATE and 2.0–16.0 Gy/GBq for [^90^Y]Y-DOTATOC. Concurrent infusion of an amino-acid solution (e.g., combination of lysine and arginine) with PRRT nowadays reduces the absorbed kidney dose as this mixture limits retention in the proximal renal tubules [21–23].

The randomized-controlled phase III NETTER-1 trial proved the efficacy of [^177^Lu]Lu-DOTATATE plus long-acting SSA in patients with metastatic GEP-NETs, and its pivotal role in treatment was solidified. Patients with inoperable, well-differentiated midgut NETs (grade 1–2) with positive lesions on [^111^In]In-pentetreotide were randomized to receive either four cycles of 7.4 GBq [^177^Lu]Lu-DOTATATE every eight weeks plus concomitant long-acting octreotide (30 mg), versus high-dose of long-acting octreotide (60 mg) every four weeks. In the primary analysis, median progression-free survival after 20 months was significantly prolonged in patients treated with [^177^Lu]Lu-DOTATATE with a hazard ration (HR) of 0.18 (95% CI 0.11–0.29; *p* > 0.0001) [24]. Final analysis after 5 years (median follow-up > 76 months) showed a median survival of 48 months in the PRRT-group (*n* = 116) and 36.3 months in the control arm (*n* = 113), with a HR of 0.84 (95% CI 0.60–1.14; *p* = 0.30) [5•]. The longer follow-up did not translate into a significant overall survival difference, however, these outcomes are impacted by the high rate (36%) of crossover of patients from the control group towards the PRRT-group after disease progression [5•, 24]. Still, it can be concluded that PRRT with [^177^Lu]Lu-DOTATATE does yield clinical benefit with regard to controlling tumor volume and improving quality of life of patients, with a confirmed low risk of hematological and renal toxicities.

The NETTER-2 phase 3 trial, a continuation on the NETTER-1 trial, which compared [^177^Lu]Lu-DOTATATE plus SSA as a first-line treatment in patients with advanced, well-differentiated GEP-NET (grade 2–3) versus high-dose octreotide alone, has recently been presented at ASCO GI 2024 [25]. The median progression-free survival was significantly prolonged by ~ 14.3 months in the PRRT-group (HR 0.276; 95% CI 0.18–0.42; *p* < 0.0001). The response rate was 43% in the PRRT arm, compared to 9% in the control arm [26••]. While long-term follow-up data are awaited, this study can potentially change the clinical practice and further broaden the use of PRRT in patients with advanced NET, especially as an earlier treatment line.

The selection of patients eligible for PRRT is based on the so-called theranostic principle, which includes imaging of the same or comparable molecule target that will be used for PRRT [27]. In this way, only patients with sufficient SSTR-expression (e.g., higher than healthy liver uptake, known as the Krenning-score) on diagnostic imaging are selected [28, 29]. Additionally, patients should have ECOG performance status < 2 and sufficient bone marrow, renal, and liver function in order to safely receive four cycles of PRRT [6]. More details on clinical implementation of the treatment can be found in the recent guidelines [1, 23].

## PRRT Combined with Transarterial Radioembolization

Majority of NET patients, up to 85%, will develop liver metastases [30]. As the foremost prognostic factor for survival and hormone-related symptoms, improved treatment of liver metastases may prolong survival but also reduce tumor related side-effects and consequently provide a better quality of life. In case of liver-only or liver-dominant metastatic disease, local radionuclide treatment using microspheres could be considered. Selective internal radiation therapy (SIRT), or transarterial radioembolization (TARE), is a safe and effective treatment in liver malignancies. It involves transarterial infusion of radioactive microspheres leading to localized irradiation [31]. NET liver lesions are generally hypervascular, causing preferential arterial flow towards tumors. This characteristic causes microspheres to lodge in tumor arterioles, ensuring high tumor accumulation of the infused microspheres, while relatively sparing normal liver parenchyma from irradiation [32]. Currently, three commercially available radioactive microspheres are used for radioembolization: ^90^Y-labeled glass spheres (Theraspheres, Boston Scientific), ^90^Y-labeled resin spheres (SirSpheres, SIRTex), and ^166^Holmium (^166^Ho)-labeled PLLA-MS spheres (QuiremSpheres, Quirem Medical). The entire work-up for radioembolization is beyond the scope of this review but has previously been explained [32].

SIRT is utilized in patients with NET either as a debulking treatment (independent of treatment line) or it is reserved as a salvage treatment following failure of other (systemic) therapies [30, 33]. SIRT is most effective when a patient-tailored dosing approach is applied (i.e., prospective dosimetry), as evident dose–response relationships are presented in literature [34, 35]. There is a difference to PRRT, where application of prospective dosimetry/patient-tailored dosing is not common. A tumor receiving a minimal mean absorbed-dose of 120 (^166^Ho)–150 (^90^Y glass) Gy has a high likelihood (> 80%) of an objective response according to RECIST 1.1. Reported local response rates for SIRT in patients with liver-only or liver-dominant disease vary between 23 and 64% (objective response as per RECIST 1.1) but are generally more profound compared to PRRT. These large variations in response rates are explained by patient, tumor, and dosing heterogeneities within studies [36].

**Fig. 1:**
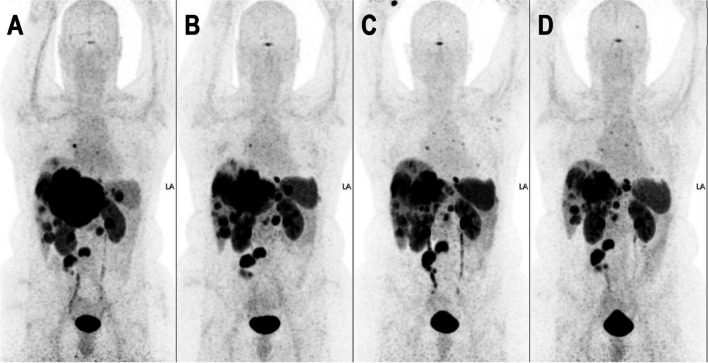
A 45-year-old, female patient with a grade 2 (ki67 7%) small intestinal NET (ileal origin), with a mesenteric mass, lymph node metastases and extensive bulky liver disease. Refractory to first-line SSA (continues flushing and diarrhea), referred for PRRT. **A** baseline [^68^ Ga]Ga-DOTATOC PET/CT. **B** [^68^ Ga]Ga-DOTATOC PET/CT 4 months after 4 cycles of PRRT, 7.4 GBq/cycle, with limited tumor reduction (RECIST 1.1 stable disease) and no improvement of complaints. 6 months after last PRRT cycle, received additional sequential whole liver ^166^Ho-SIRT. **C** [^68^ Ga]Ga-DOTATOC PET/CT 3 months after additional ^166^Ho-SIRT, additional tumor reduction (partial response according to RECIST 1.1) and resolution of flushing and diarrhea (with concurrent long-acting SSA, additional short-acting SSA was stopped). **D** [^68^ Ga]Ga-DOTATOC PET/CT 20 months after ^166^Ho-SIRT, showing durable response, with continues decrease of bulky liver disease (hepatomegaly has clearly been reduced). No signs of ‘pseudo-cirrhotic’ morphology on anatomical imaging were observed ever since

As illustrated by the post-hoc analysis of the NETTER-1 trial [37], progression free survival (PFS) following PRRT is significantly shorter in patients suffering from “bulky” liver disease (defined as having a lesion > 3 cm maximum diameter), which was present in approximately 70% of patients. Furthermore, patients can experience residual hormone related complaints following PRRT, assumed to be caused by liver metastases, bypassing the “filtration” of the liver. Therefore, combining systemic and local radionuclide treatments seems logical, gaining systemic control with PRRT whilst reducing prognostic significant liver disease (Fig. 1).

Based on retrospective data and results from the prospective phase 2 HEPAR PLuS study, it can be concluded that radioembolization and PRRT (4 cycles of 7.4 GBq [^177^Lu]Lu-DOTATATE) can safely be used in the same patient [38, 39]. In general toxicities were minor and transient, but the most commonly reported grade 3–4 toxicities at six months included abdominal pain (10%), lymphocytopenia (23%), and increased gamma-glutamyl transpeptidase (54%). In the HEPAR PLuS study, hepatic and patient-based response was over 40% at three months, which is high compared to the 18% achieved in the NETTER-1 study. Even with this prospective data, there is no broad clinical implementation of PRRT plus radioembolization, due to some fear of anecdotal reports of long-term hepatotoxicity when combining SIRT with systemic treatments [40]. However, evidence for late hepatotoxicity is scares, so long-term follow-up in these patients is needed.

## PRRT Combined with Chemotherapy

The current role of chemotherapy in patients with grade 1–2 NETs is limited, because cytotoxic chemotherapies are most effective in malignancies that are more rapidly proliferating. According to ENETS guidelines, chemotherapy is recommended for progressive or advanced (bulky) pancreatic NET (pNET), grade 3 neuroendocrine neoplasms (NENs), and neuroendocrine carcinoma (NEC) [41]. Nevertheless, chemotherapy is widely used as a radiosensitizer during external-beam radiotherapy (EBRT) for various malignancies. Radiosensitizers like platinum, gemcitabine, and fluoropyrimidines are utilized to enhance radiation-induced cellular damage by suppressing radioprotective molecules, inhibiting DNA-repair mechanisms, or dysregulating the cell cycle [42].

Combinations of PRRT and chemotherapies are predominantly explored in phase 1 studies. A notable limitation in the existing literature lies in the diverse dosing regimens and chemotherapeutic agents, with most evidence focusing on capecitabine (CAP), temozolomide (TEM), and 5-fluorouracil (5-FU) combined with various PRRT-ligands. Claringbold et al. conducted a phase 2 study treating patients with advanced progressive pNETs with [^177^Lu]Lu-DOTATATE (four cycles ~ 7.9 GBq) combined with capecitabine (1500 mg/m^2^; 14 days) and temozolomide (200 mg/m^2^; 5 days) each cycle [43]. The overall response rate was 80%, with complete remission achieved in 13%; median overall survival (OS) has not yet been reached at 33 months follow-up. This combination was well tolerated, with the main toxicities being nausea (40%, grades 2–3) and hematological toxicity (10%, grades 2–3). In a prospective study by Nicolini et al. (2021), 37 patients with FDG- and SSTR-positive NETs were treated with five cycles of 5.5 GBq PRRT combined with capecitabine alone (1000–1500 mg/d) [44]. No significant (> grade 2) toxicities were observed during the median 30 months follow-up. A partial response was seen in 30% of patients, and median PFS reached 31.4 months. The effect of concomitant chemotherapy (600 mg/m^2^/d capecitabine and 75 mg/m^2^ temozolomide) with ~ 7.4 GBq [^177^Lu]Lu-DOTATATE on physiological uptake and tumor accumulation was assessed in 20 patients with advanced GEP-NET [45]. Those receiving combination treatments had comparable ^177^Lu-accumulation in tumor lesions (3.85 ± 1.74 mGy/MBq versus 5.6 ± 11.27 mGy/MBq) compared to the PRRT-alone group. Also, no difference in kidney, liver, spleen, and bone marrow uptake was observed.

While there is a growing body of research on this topic, it has yet to provide conclusive evidence supporting the observed synergistic effect, when combining radiotherapy and chemotherapy. Extensive literature reviews conducted by Chan et al. [46•] and Santo et al. [47] regarding this specific topic concluded have underscored the necessity for additional prospective data to delineate the efficacy and safety of PRRT in conjunction with chemotherapy regimens, before wide-spread clinical adaptation becomes feasible. Presently, several ongoing prospective (randomized controlled) clinical trials are investigating the effect of PRRT plus capecitabine (with or without temozolomide), additional details are outlined in Table 2.

**Table 2:** Current clinical trials investigating PRRT combination therapies

NCT	Trial ID	Origin	Est. enrollment	Combined with	PRRT	Dosage	Study phase	Status
04194125		Poland	25	Chemotherapy; CAPTEM	[^177^Lu]Lu -DOTATOC	Up to 4 cycles 7.4 GBq	II	Unknown
05387603	START-NET	Sweden	300	Chemotherapy; capecitabine	[^177^Lu]Lu -DOTATOC	Up to 7 cycles 7.5 GBq	III	Not yet recruiting
02736448	Lu-Ca-S	Italy	35	Chemotherapy; CAPTEM	[^177^Lu]Lu-PRRT	7 cycles 3.7 GBq	II	Unknown
05053854	PARLuNET	Australia	24	Targeted therapy; PARP inhibitor/talazoparib	[^177^Lu]Lu-DOTATATE	4 cycles, dose unknown	I	Recruiting
05249114		Oregon, USA	6	Targeted therapy; cabozantinib	[^177^Lu]Lu-DOTATATE	4 cycles 7.4 GBq	I	Active, not recruiting
05870423		The Netherlands	24	Targeted therapy; PARP inhibitors; olaparib	[^177^Lu]Lu-DOTATATE	Several cycles 7.4 GBq	I	Recruiting
04375267	LuPARP	Sweden	18	Targeted therapy; olaparib	[^177^Lu]Lu-DOTATATE	4 cycles, unknown dose	I	Active, not recruiting
04543955		Kentucky, USA	70	Targeted therapy; telotristat	[^177^Lu]Lu-DOTATATE	4 cycles, unknown dose	II	Unknown
04750954		Multicenter, USA	29	Targeted therapy; M3814, peposertib	[^177^Lu]Lu-DOTATATE	4 cycles, unknown dose	I	Recruiting
05687123		California, USA	24	Targeted therapy; sunitinib	[^177^Lu]Lu-DOTATATE	4 cycles, unknown dose	I	Recruiting
05724108		Multicenter, USA	94	Targeted therapy; triapine	[^177^Lu]Lu-DOTATATE	4 cycles, unknown dose	II	Recruiting
05178693	LANTana	UK	27	Targeted therapy; ASTX27	[^177^Lu]Lu-DOTATATE	4 cycles, 7.4 GBq	I	Recruiting
04234568		Multicenter, USA	31	Targeted therapy; triapine	[^177^Lu]Lu-DOTATATE	4 cycles, 7.4 GBq	I	Abstract published
04086485		Maryland, USA	42	Targeted therapy; olaparib	[^177^Lu]Lu-DOTATATE	4 cycles, unknown dose	I/II	Recruiting
03457948		California, USA	32	Immunotherapy; pembrolizumab	[^177^Lu]Lu-DOTATATE; ^90^Y microsphere radioembolization	Up to 4 cycles, unknown dose	II	Active, not recruiting
04525638		Spain	30	Immunotherapy; nivolumab	[^177^Lu]Lu-DOTATATE	Up to 4 cycles,7.4 GBq	II	Recruiting
03044977		Iowa, USA	20	Double PRRT	[^90^Y]Y-DOTATOC and [^131^I]I-MIBG	Up to 2 cycles, customized doses	I	Active, not recruiting
04385992	NeoLuPaNET	Italy	31	Neoadjuvant PRRT	[^90^Y]Y-DOTATOC and [^177^Lu]Lu -DOTATATE	4 cycles, unknown doses	II	Abstract published
04614766	SPORE-3	Iowa, USA	50	Double PRRT	[^131^I]I-MIBG + [^177^Lu]Lu-DOTATATE	4 cycles, unknown doses	I/II	Recruiting

## PRRT Combined with Targeted Therapy

Everolimus is currently standard of care treatment for patients with advanced pNETs or progressing on prior lines of therapy. It is an inhibitor of mTOR, which stimulates cell growth, proliferation, and angiogenesis. Two phase I studies were published that analyzed safety and efficacy with a combination of everolimus and PRRT. The first study from 2015 included 16 patients with advanced unresectable progressive well-differentiated GEP-NETs [48]. They received four cycles of 7.8 GBq [^177^Lu]Lu-DOTATATE at eight-week interval and the dose of everolimus was escalated (5–7.5–10 mg daily for 24 weeks). They found an overall response rate of 44% and a maximum tolerated dose of 7.5 mg. The second trial treated three patients with 5 mg and the next eight patients with 10 mg everolimus daily [49]. The regular dosage of 10 mg everolimus daily was not tolerated, indicating that a lower dose is warranted in this combination therapy.

Sunitinib inhibits a variety of receptor tyrosine kinases involved in tumor growth, pathological angiogenesis and progression of cancer, and is one of the targeted therapies that provided new treatment opportunities in patients with pNETs. Although there is some evidence of a synergistic effect between radiotherapy and sunitinib in other malignancies [50], data in NETs remains limited. Another potential combination with PRRT is the targeted Poly (ADP-ribose) polymerase (PARP) inhibitor. PARP is an enzyme that plays a vital role in the repair of damaged DNA. Pre-clinical and theoretical work has shown the potential of combining PARP inhibitors and PRRT to enhance cell death and overall survival in NET cell-lines [51–53]. Human studies are still in the early stages. Ongoing clinical trials investigating the combination of targeted therapies with [^177^Lu]Lu-DOTATATE are also outlined in Table 2.

## PRRT Combined with Immunotherapy

Radiation not only kills tumor cells, but it can also trigger some anticancer immune responses by increasing tumor antigen release and promoting immune cell infiltration. In contrast, immunotherapies work by directly activating the body’s immune system to recognize and attack cancer cells more effectively. The combination of these treatments may act synergistically to generate antitumor immunity and enhance overall therapeutic efficacy [54].

Immune-checkpoint-inhibitors, such as nivolumab and pembrolizumab (both anti-PD-1 antibodies), are considered groundbreaking in other solid tumors, but for NET limited data are available. The feasibility of combining nivolumab with [^177^Lu]Lu-DOTATATE was assessed in a phase I dose-finding study in nine patients with lung-NET [55]. Administered activities of 3.7 and 7.4 GBq [^177^Lu]Lu-DOTATATE every eight weeks (four cycles) were combined with four doses nivolumab (240 mg every two weeks). No dose-limiting toxicities were observed at 3.7 GBq, but at 7.4 GBq one patient developed a grade 3 rash. Other adverse events included lymphopenia (*n* = 7), thrombocytopenia (*n* = 4), anemia (*n* = 3), and nausea (*n* = 3). An overall response rate of 14.3% was found. Other clinical ongoing studies are outlined in Table 2.

## The Role of PRRT Prior to Surgery

Several retrospective analyses suggest the potential efficacy of PRRT prior to surgery [56, 57]. Partelli et al. (2018) retrospectively compared two cohorts with pNET; one receiving PRRT before surgery, either [^177^Lu]Lu-DOTATATE or [^90^Y]Y-DOTATOC, and the other group underwent surgery without prior PRRT. Patients in the first group received various dose schedules of PRRT; either five cycles of 5.5 GBq (750 mCi total) or eight cycles of 3.7 GBq (800 mCi total) of [^177^Lu]Lu-DOTATATE, or either four cycles of 2.8 GBq (300 mCi total) or four cycles of 1.85 GBq (200 mCi total) of [^90^Y]Y-DOTATOC. Although the study was limited in size with 23 patients in each group, it demonstrated a reduced incidence of nodal metastases (9/23 vs 17/23; *p* < 0.02) and lower risk of pancreatic fistulas in the PRRT group (0/23 vs 4/23; *p* < 0.02). It did not find a significant difference in PFS (52 vs 37 months; *p* > 0.2) [58]. Similarly, Parghane et al. investigated the administration of four to five cycles 7.4 GBq [^177^Lu]Lu-DOTATATE prior to surgery in a heterogeneous population of 57 patients with GEP-NETs. They observed that 26% of patients achieved resectability of the primary tumor post-PRRT, with notable PFS rates at median follow-up of 24 months of 95% and 90%, either without (*n* = 23) or with liver metastases (*n* = 34), and 2 year OS of both groups combined was 92% [59].

The most recent study on PRRT before surgery by Minczeles et al. compared OS of 23 patients with pNETs with PRRT-only and 26 patients with PRRT followed by surgery [60]. They found an average decrease in pNET size of 26% (RECIST 1.1) and a reduction of vascular involvement. Total median OS was 8.5 years (95%, 4.5–12.5 years), with the cohort receiving surgery + PRRT exhibited a median OS of 14.7 years (95%, 5.9–23.6), compared to 5.5 years (95%, 4.5–6.5) for the PRRT-only group (*p* = 0.003). However, it is noteworthy that baseline comparability between the groups was not fully achieved, as the surgery cohort demonstrated a shorter interval from diagnosis to treatment initiation, alongside a notably higher proportion of grade 1 NETs. These findings underscore the potential benefits of presurgical PRRT in improving surgical outcomes and patient prognosis.

## Future Directions

The theranostic approach is the foundation for PRRT, as imaging plays an eminent role in patient selection, treatment verification and follow-up. Prior to PRRT, SSTR-targeted imaging can help to assess SSTR-status of lesions, quantify tumor burden, and provide details on whole-body tracer distribution. Imaging during therapy can be used to measure the absorbed radiation dose in NET lesions and organs at risk (e.g., liver, bone marrow, and kidneys). While for radioembolization it has been proven that image-based treatment planning results in a more effective therapy with a lower chance of side-effects, this approach is not the standard for systemic PRRT. The goal of image-based planning is to estimate the therapeutic dose distribution, and modify the administered activity accordingly to increase the absorbed dose in the tumor, while controlling the absorbed dose in healthy tissues. This approach is an elaborate balance between the tumor control probability and normal tissue complication probability.

A dose–response relationship has been described for PRRT, but the scientific evidence is limited to observations in clinical cohorts and retrospective studies. Additionally, the limited implementation of personalized dosing is related to the fact that [^177^Lu]-DOTA-0-Tyr3-Octreotate (Lutathera®) are registered based on a fixed-dose posology. So any modifications in administered activity or treatment schedule could be considered off-label use. Exceptions are suggested in the product registration in cases where there is an increased risk of hematological toxicity (e.g., in patients with high skeletal metastatic burden), hepatotoxicity (e.g., in patients with high hepatic tumor load), or nephrotoxicity.

In the context of metastatic GEP-NET, therapeutic efficacy of PRRT as a mono-therapy could be improved by increasing the administered activity in the first two cycles, as the receptor density on tumors decreases over treatment cycles as a result of this therapy [61•, 62, 63]. More elaborate dose modifications in PRRT involve image-based treatment modelling either using the diagnostic SSTR-PET or therapeutic SPECT. The absorbed radiation dose to the tumor and organs-at-risk is calculated, and the administered activity for the subsequent cycles can then be adapted accordingly. Though image-based treatment planning for PRRT is feasible in a routine clinical practice, it is considered labor intensive by many and there is no consensus yet on effective absorbed dose levels.

In patients with low SSTR-expression whom are either considered ineligible for PRRT or respond poorly to [^177^Lu]Lu-DOTATATE, combinations with radiation sensitizers, DNA-repair inhibitors or immune-activating agents could improve treatment efficacy. There are also new molecular targets that may hold an even greater promise in NET and other malignancies. In NENs and related malignancies, fibroblast-activation protein (FAP) and urokinase plasminogen activator receptor (uPAR) are interesting targets for radionuclide therapy as they show expression in both low-grade and high-grade tumors. The first dose escalation studies to demonstrate the usability of Lutetium-labeled FAP-inhibitors in patients with NET are currently recruiting (NCT05432193, NCT04459273). In addition to SSTR-agonists, also antagonists have been opted as effective target.

In the search for more effective therapies, attention has shifted from beta-emitting towards alpha-emitting nuclides such as Actinium-225 (^225^Ac) and Lead-212 (^212^Pb). The advantage of alpha-radiation is that it has a high energy transfer upon interaction with tissues, and thus, has the ability to induce a lot of damage with a low range. At present, [^225^Ac]Ac-DOTATATE is the leading alpha-emitting radiopharmaceutical and mainly used in patients who progress after [^177^Lu]Lu-DOTATATE therapy. Though in certain cases remarkable responses have been described, the evidence for [^225^Ac]Ac-DOTATATE in patients with NET is still limited to small scale clinical studies in selected patient cohorts. Currently, after concluding its phase 1 part, the phase 3 randomized controlled trial ACTION-1 is recruiting patients in North America and Europe for patients with advanced NET, after initial [^177^Lu]Lu-DOTATATE therapy (NCT05477576). As with many developments in radionuclide therapies, most are implemented into “compasionate use” programs to gain the evidence needed to warrant subsequent phase I-II studies.

## Availability of PRRT

As [^177^Lu]Lu-PRRT is nowadays considered a proven mono-therapy in patients with metastatic GEP-NET, the use of these therapies is increasing worldwide. Although [^177^Lu]Lu-DOTATATE was traditionally performed in specialized institutions, commercialization of PRRT has enabled smaller hospitals and day-clinics to also perform these therapies. This increased accessibility is considered an advantage for patients, but there are also concerns raised regarding their clinical benefit versus costs and general availability [33, 64]. Due to the increasing global demand, access to therapeutic radiopharmaceuticals is not always ascertained, so efforts are made to improve supply chains [65]. So, it has been postulated by ENETS and EANM that oncological radionuclide therapies should be mainly conducted, or at least coordinated, by specialized centers.

## Conclusion

Following the publication of the NETTER-1 trial, which underscored the relevance of [^177^Lu]Lu-DOTATATE in the treatment of patients with advanced NET, extensive research has been conducted to further improve the treatment. This includes the potential synergistic combination of PRRT with other established treatments for patients with NET. Various treatments have been combined with PRRT, but evidence on the benefit of combined treatments is limited. Nonetheless, there are numerous ongoing clinical trials aiming to define the synergistic effect further.

## Data Availability

No datasets were generated or analyzed during the current study.
